# Supplementary material for: The Effect of Natural-Based Formulation (NBF) on the Response of RAW264.7 Macrophages to LPS as an In Vitro Model of Inflammation
Source: J Fungi (Basel). 2022 Mar 21;8(3):321. doi: 10.3390/jof8030321 (PMC8955716; doi:10.3390/jof8030321)
Supplement: Supplementary file 1 [file jof-08-00321-s001.zip › Figures S1 and S2.pdf]

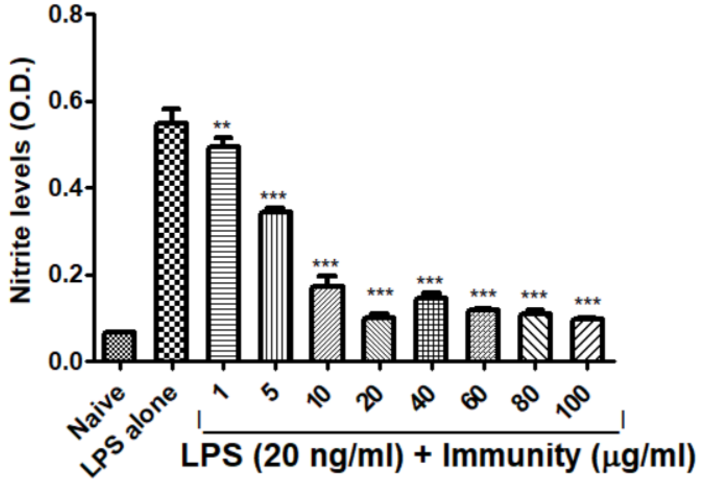

Supplementary Figure S1. Dose-response of nitrite accumulation (NO release) following exposure of RAW 264.7 macrophages to LPS (20 ng/ml) in the absence or presence of various concentrations of Immunity (µg/ml). One way ANOVA was performed with Bonferroni's post-hoc correction (n=4 for each group). ** p<0.01 and *** p<0.001 compared to LPS alone.

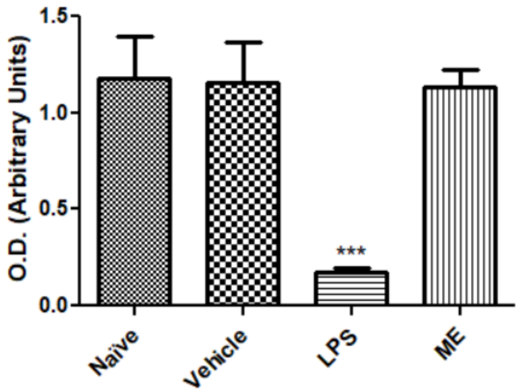

Supplementary Figure S2. XTT assay of RAW264.7 cells following 72 hours treatment with mushroom extract. The RAW264.7 cells were exposed to 20 ng/ml LPS along with or without corresponding vehicle and mushroom extracts. The 20 µg/ml of mushroom extract did not show any toxic effects until 72 hours of the treatment. One way ANOVA was performed with Bonferroni's post-hoc correction (n=8 for each group). *** p<0.001 compared to naïve/vehicle.
