# Supplementary material for: The Effect of Natural-Based Formulation (NBF) on the Response of RAW264.7 Macrophages to LPS as an In Vitro Model of Inflammation
Source: J Fungi (Basel). 2022 Mar 21;8(3):321. doi: 10.3390/jof8030321 (PMC8955716; doi:10.3390/jof8030321)
Supplement: Supplementary file 1 [file jof-08-00321-s001.zip › File S1.pdf]

Supplementary data for Figure 6:
Primers:
TLR2-Mus-F: 5' – AAGAGGAAGCCCAAGAAAGC - 3'

TLR2-Mus-R: 5' – CAATGGGAATCCTGCTCACT - 3'

Product size: 80bp

TLR4-Mus-F: 5' – GGCAGCAGGTGGAATTGTAT- 3'
TLR4-Mus-R: 5' – AGGATTCGAGGCTTTTCCAT- 3'
Product size: 131bp
NF-κB -F: 5' – CACCTAGCTGCCAAAGAAGG- 3
NF-κB -R: 5' – GCAGGCTATTGCTCATCACA - 3
Product size: 136 bp

GAPDH-Mus-F: 5' – CCTGCACCACCAACTGCTTA- 3
GAPDH-Mus-R: 5' – CCATCCACAGTCTTCTGAG - 3'

Product size: 131 bp
